# Different Drying Techniques and Their Impact on Physicochemical Properties of Sweet Potato: A Review

**DOI:** 10.1111/1750-3841.70458

**Published:** 2025-08-11

**Authors:** Khuthadzo Ntsowe, Tilahun Seyoum Workneh, Sunette Laurie, Naushad Emmambux

**Affiliations:** ^1^ Bioresources Engineering, School of Engineering University of KwaZulu‐Natal Pietermaritzburg South Africa; ^2^ Agricultural Research Council‐Vegetables, Industrial and Medicinal Plants Roodeplaat Pretoria South Africa; ^3^ Department of Consumer and Food Science University of Pretoria Pretoria South Africa

**Keywords:** combined drying methods, drying techniques, drying factors, physicochemical properties, sweet potato

## Abstract

Sweet potato (SP) is a globally important storage root crop, serving as a natural source of essential nutrients, including starch, carotenoids, and anthocyanins. Given its nutritional value, preserving the physicochemical properties during processing is imperative to enhance its role in addressing food security. This review presents a comprehensive analysis of the literature on physicochemical properties, such as color, β‐carotene, and TPC and examines the effects of different drying techniques on these properties. It was found that CHAD significantly degrades these properties, potentially reducing the β‐carotene content by up to 22.7‐fold and the TPC by 53%. This degradation can result from high drying air temperatures (>70°C), high air velocity (>1.5 m.s^−1^) and extended drying times, which can be up to 28 h. In contrast, combined techniques preserve these properties. For example, MWD + CHAD reduced the drying time up to six times more than CHAD alone. In addition, MWD + CHAD achieved a lower total color change (ΔE) and a higher retention of β‐carotene content (67%). The highest increase in TPC (247%) was observed for SP that was dried using MWD with carbon maceration pre‐drying treatment. This results from a porous microstructure that is formed which increases the moisture transfer and reduces the drying time. Factors like the drying air temperature, drying medium, air velocity, and pre‐drying treatments influence the performance of drying techniques and their quality preservation capabilities. However, the SP variety is often overlooked in drying studies. This article discussed, compared, and identified literature gaps to pave the way for future research aimed at enhancing dried SP product quality.

## 1 Introduction

**FIGURE 1:**
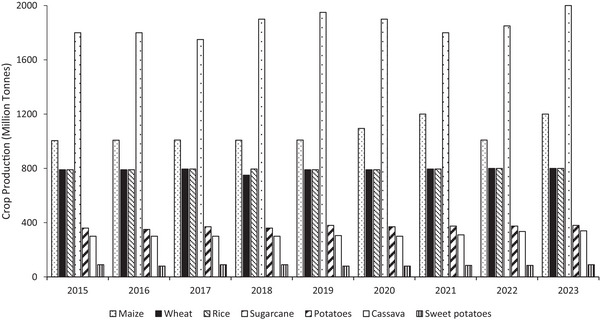
Top produced crops globally as extracted from (FAO 2024).

**FIGURE 2:**
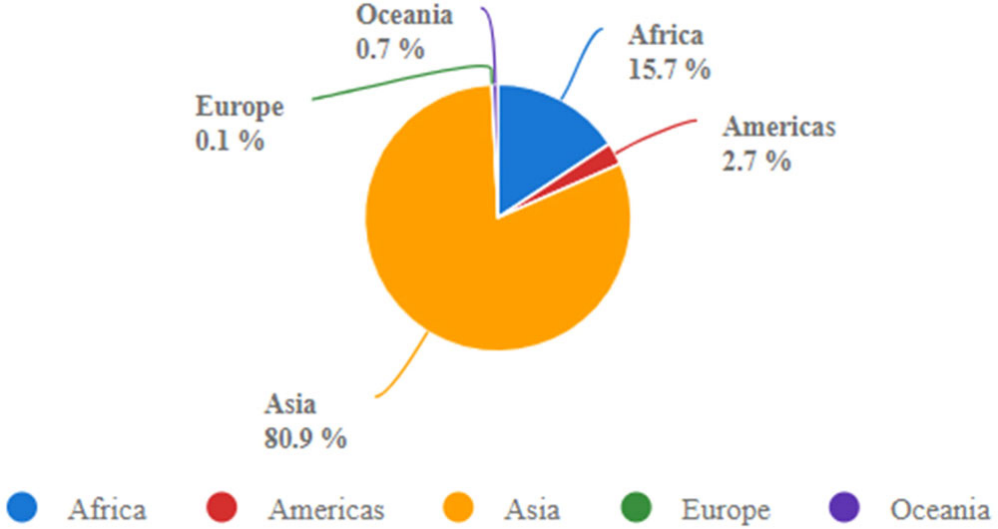
Regional share of SP production (FAO 2024).

Sweet potato *(Ipomoea batatas L.) sto*rage root is an important root tuber crop globally. It is the seventh most produced food crop after maize, rice, wheat, sugar crops, potato, and barley (Figure 1). SP has a potential to meet the nutritional needs of developing nations (Laurie et al. 2023; Islam 2024). Therefore, sweet potatoes have assumed the status of food security crops in many countries (Sugri et al. 2024), due to their high starch with low glycemic index, anthocyanin, carotenoids, and minerals, adaptability to various agroclimatic conditions and low production costs (Hossain et al. 2022; Islam 2024). In 2023, the global SP production reached 93.5 million tons. China and Africa were the leading producers, contributing 80.9 % and 15.7% of the total, respectively (figure 2) (FAO 2024). According to Laurie et al. (2022), “the major contribution of SP is through pro‐ vitamin A biofortified varieties to alleviate malnutrition.” Thus, SP's are vital for achieving Sustainable Development Goal 2.

Despite its global significance, the commercial use of SP, particularly in value‐added products has been limited. This includes shelf‐stable flours, which are key ingredients in functional foods and healthy snacks (Sapakhova et al. 2023; Gurmu et al. 2024). The benefits of SP‐based products include improved color, flavor, supplemented nutrients, and natural sweetness. Recent reviews on SP storage roots covered the associated health benefits of SP and its varieties (Alam 2021; Rosell et al. 2024), phytochemical constituents, biological activities and effects of processing (Laveriano‐Santos et al. 2022), 72 years of SP research in South Africa (Laurie et al. 2023), and sweet potatoe's potential as a sustainable and versatile food source for future generations (Islam 2024). Considering the existing research, it was evident that further understanding of the physicochemical changes induced by drying is essential for advancing SP‐based product formulation.

Drying is a primary step used in processing of SP to create shelf‐stable flour that is used for value‐added purposes in food products. This process minimizes the chemical and physical changes that occur during storage by reducing the water content and microbial activity, thereby enhancing SP's stability (Marzuki et al. 2021; Jiang et al. 2025; Yildiz et al. 2025). Given the nutritional and commercial value of SP in food processing, optimizing drying techniques is crucial for preserving the quality. Conventional methods, whilst simplistic and cost‐effective, often compromise the physicochemical properties of SP (Bhattacharjee et al. 2024). Generally, heat treatments have a negative effect on the properties of SP (Guclu et al. 2023). For example, CHAD was reported to reduce the β‐carotene content of SP between 43% and 60% (Ahmed et al. 2010). It also causes darkening of SP (Savas 2022), and a cracked microstructure (Onwude et al. 2018). Previous studies have confirmed that the ability of a drying technique to preserve quality depends on the drying air temperature, air velocity, slice thickness, and pre‐drying treatment used (Wang et al. 2020; Gasa et al. 2022a; Jiang et al. 2025). Many studies have independently investigated these factors and the physicochemical properties of SP. However, there is a lack of collated information on SP drying and their effects on physicochemical properties, with consideration of emerging drying techniques. Hence, this study aims to provide a comprehensive analysis of literature by integrating and updating information on SP drying techniques as well as their impact on physicochemical properties.

## 2 Physicochemical Properties of Sweet Potatoes

Physicochemical properties encompass both the physical (color, texture, weight, viscosity) and chemical properties (proximate composition, mineral content, and antioxidant assays). These properties often form the basis for SP exploitation (Vithu et al. 2020). Common SP varieties include white‐ (WFSP), cream‐ (CFSP), yellow‐ (YFSP), orange‐ (OFSP), and purple‐fleshed (PFSP). Generally, SP is low in protein (2.48 ‐6.50 g.100g^−1^), due to limiting amino acids, methionine and cysteine, which restrict the biological value of SP protein by limiting overall protein synthesis (Hong et al. 2020; Hou et al. 2020). Additionally, it is considered a low‐fat product, with a fat content that is less than 3 g.100g^−1^ (Table S1), and it is rich in starch, moisture, and dry matter content (Hossain et al. 2022), making it an ideal crop for processing. Its low paste viscosity and digestibility, make it a suitable thickening agent (Dereje et al. 2020) and ideal for infant foods (Amagloh 2022). The physicochemical properties of fresh SP vary significantly depending on the genetic composition, agroclimatic conditions, and the breeding goals of regions, as shown in Table S1 (superscripts). For instance, the OFSP variety, Bophelo, contain up to 12% protein, whereas the WFSP Blesbok contains only 4% (Ayeleso et al. 2024). Similarly, with the dietary fiber, Ngcobo et al. (2024) found that among PFSP genotypes, the Brazilian had lower dietary fiber than the South African one, while the Chinese genotypes showed higher levels.

Historically, breeding goals in developing countries, including those in Africa, focused on high dry matter SP, due to their elevated carbohydrate levels (energy value of 446 KJ.100g^−1^), compared to other staples (Laurie et al. 2018; Islam 2024). However, recent years have seen increased research efforts on OFSP, YFSP, and PFSP, owing to their high levels of carotenoids (e.g., β‐carotene), phenolics, and anthocyanins, with their flesh color being linked to the health effects (Vithu et al. 2020; Alam 2021; Laveriano‐Santos et al. 2022). For example, β‐carotene levels in SP varieties have shown an upward trend since 1999. While typical values for OFSP range from 6.7 to 13.1 mg.100 g^−1^, the Korean varieties have higher values of 530 mg.100 g^−1^ (Kim et al. 2015). Kim et al. (2015) demonstrated that PFSP exhibited a higher level of antioxidant activity, than OFSP and WFSP varieties. Therefore, selecting the appropriate variety to maximize the nutrient bioaccessibility, which can potentially increase or decrease depending on the drying method, is a crucial consideration in dryer optimization.

## 3 Sweet Potato Drying Techniques

**FIGURE 3:**
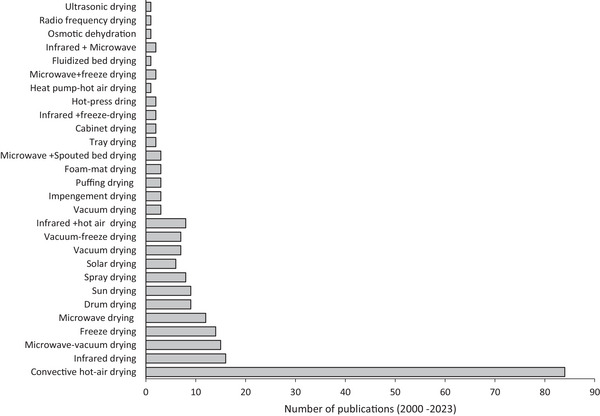
Trends in drying technologies for SP for publications from 2000 to 2023 (Scopus).

Conventional drying techniques, including convective hot‐air drying (CHAD), freeze drying (FD), spray drying (SD), vacuum drying (VD), and infrared drying (IRD) are widely used in SP processing. A study performed using the Scopus database for the period 2000–2023 with the search terms “SP” and “drying” revealed that CHAD is the common drying technique, with 84 publications (Figure 3). The limitations of individual drying techniques like CHAD include prolonged drying periods, lower drying rates, high energy consumption, and non‐uniform moisture distribution, which negatively impact the product quality (Onwude et al. 2018). Therefore, researchers have explored integrating conventional techniques, such as infrared + vacuum drying (12 publications), hot‐air + IRD (8 publications), and microwave + vacuum drying (12 publications) to enhance the drying process. Hence, this summary includes some of the drying techniques that have been used in drying SP, as shown in Table 1.

**TABLE 1:** Summary of drying methods used in drying sweet potatoes.

Drying technique	Sweet potato variety	Sample type (size)	Process conditions and pre‐drying treatments	Key highlights	References
OSD	NR	Slices	T = 20 – 41°C, RH = 19 – 52%, Solar radiation = 269 – 354.5 W.m^−2^	‐ Solar drying duration:31 h to 7 days. ‐ Moisture loss per day was about 7%. ‐Fungal (91%) and bacterial (100%) contamination have been reported.	Onyenwigwe et al. (2023); Watson et al. (2024)
SD	OFSP, most studies did not report the variety used.	Slices (3 ‐7 mm), Cubes (NR)	Ambient conditions: T = 10 ‐ 41°C, RH = 19 ‐97%, v = 0.8 ‐ 9.7 m.s^−1^, solar radiation = 269 – 858 W.m^−2^, sunshine hours = 2.2 – 9 h Drying chamber conditions: T = 21 ‐ 63°C, thermal efficiency = 29.9 – 97.2% Pre‐drying treatments: Blanching, lemon juice, iodated table salt	‐ Solar drying durationvaried between 11 and 57 h, depending on slice thickness, air velocity, temperature and RH. ‐ CO_2_ mitigation between 12 and 20.25 tons for a period of 35 years, depending on dryer simplicity. ‐ Can increase ambient air temperature between 7 and 8.2°C. ‐ Blanching results in darker color, and microstructure with deformed starch.	Abdelkader et al. (2024); Onyenwigwe et al. (2023); Sakouvogui et al. (2023); Gasa et al. (2022b);
CHAD	OFSP, PFSP, WFSP	Slices (2‐15 mm)	T = 40 – 80°C, v = 1 ‐ 4.5 m.s^−1^ Pre‐drying treatment: blanching, ultrasound, citric acid, sodium benzoate	‐ Drying duration can vary between 4 h and 28 h, depending on slice thickness, air velocity and temperature. ‐ Highest SEC was 338 kWh.kg^−1^. ‐ Blanching reduced the drying rate, whilst ultrasound reduced drying time by 100.8%. ‐ ΔE was between 14.48 – 28.99 andsignificant (*p <* 0.05) decrease in TPC was observed.	Kgonothi et al. (2024); Tayyab Rashid et al. (2022); (Moloto et al. 2021); Onwude et al. (2018); Liu et al. (2017); Falade and Solademi (2010)
CHAD + IRD, CHD, IRD	NR	Slices (4 ‐ 6 mm)	IR intensity = 1100 ‐ 1400 W.m^−2^, T = 50 – 70°C	‐ CHAD + IRD reduced the SEC of CHAD and drying time, up to 86% and 74%. ‐ There was no significant difference in ΔE between the samples dried in CHAD and CHAD+IRD.	Onwude et al. (2018)
IRD	NR	Slices (3 ‐ 8 mm)	Power = 104 ‐167 W	‐ The drying time decreased with increasing microwave power and decreasing slice thickness.	Doymaz (2012)
IRFD	NR	Slice (5 mm)	T = 50°C, Pressure = 80 Pa Pre‐drying treatment: ultrasound	‐ Ultrasound pre‐treatment increased the β‐carotene content by 42%.	Wu et al. (2020)
SBD, MSBD	PFSP	Cubes (0.1 ‐1 cm^2^)	T = 50 ‐ 120°C, microwave power = 2 ‐ 4 W.g^−1^	‐ The optimum drying temperature was found to be 70°C.	Rezende et al. (2024); Liu et al. (2014)
FD, two stage MIFD, VFD	WFSP	slices	T = 40 ‐ 60°C, vacuum pressure = 5 kPa, IR intensity = 3 ‐5 kW.m^−2^	‐ IR for 5 min at beginning of drying at 60°C prior to freeze drying had the highest reduction of drying time (52%) and the lowest energy uptake. ‐ The highest hardness was observed for VFD samples, followed by MIRFD and FD. ‐ MIRFD had the highest ΔE (23.52), followed by VFD (21.90), and FD (16.13) for untreated sweet potato.	Antal (2023)
MWFD, MWSBD	PFSP	Slices (10 mm)	MWFD: T = ‐38 – 45°C, pressure = 100 Pa, microwave power = 4 W.g^−1^ MWSBD: T = 80°C, microwave power 2.5 W. g^−1^	‐ MWFD had the longest drying time and significantly (*p* < 0.05) high SEC. ‐ ΔE was high in order of MWFD> MWVD> MWSD ‐ Anthocyanin retention was higher for MWFD (74.98%) than MWSBD (53.7%) and MWVD (71.41%).	Liu et al. (2014)
Two stage MWD + CHD	NR	Slices (2 – 4 mm)	T = 70 ‐ 80°C, microwave power = 100 ‐ 300 W	‐ Highest energy consumption at 300 W and 90°C, highest energy efficiency (82%) at 100 W and 80°C.	Sobowale et al. (2022)
SBD, MWSBD	PFSP	Cubes (0.1 ‐1 cm^2^)	T = 50 ‐ 120°C, microwave power = 2 ‐ 4 W.g^−1^	‐ The optimum drying temperature = 70°C.	Rezende et al. (2024);

CHAD = Convective hot‐air drying, FD = Freeze drying, IRD = infrared drying, IRFD = infrared freeze drying, NR = not reported, MIFD = mid‐infrared freeze drying, MVD = microwave vacuum drying, MWD = microwave drying MWSBD = microwave spouted bed drying, MWD = microwave drying, MWFD = microwave freeze drying, OSD = open air solar drying, RH = relative humidity, SBD = spouted bed drying, SD = solar drying, T = air temperature, v = air velocity, VFD = vacuum freeze drying.

**TABLE 2:** A summary of the effects of drying on physicochemical properties of sweet potatoes.

Drying technique	Pre‐drying treatments	Slice thickness (mm)	Key effects on quality parameters of sweet potatoes	References
CHAD	OD, freeze thawing, US, glucose solution	4‐10	‐ CHAD achieves visible color changes (ΔE = 34.24), lowest β‐carotene retention (40%). ‐ Freeze thawing and osmotic pre‐drying treatment reduced drying time 80% and 66.67%. ‐ TPC was significantly (*p* < 0.05) higher than untreated. ‐ Low enzymatic browning for untreated samples than US and glucose treated samples. ‐ Lower hardness for US pre‐treated samples. ‐ High concentration glucose (20% w/v) preserved color better than US.	Yan et al. (2013); Tayyab Rashid et al. (2020); Osae et al. (2024)
FD	Sodium metabisulphite	3‐5	‐ Creates a porous structure with decreased bulk density and achieves lower ΔE that is < CHAD, IRD, MWD and IRD+MWD. ‐ β‐carotene retention of FD < IRD+MWD < MW < IR < CHAD but > SJR+US. ‐ Increase in TPC < MWD but > fresh SP and CHAD. ‐ Hardness value similar to SJR+ US.	Jing et al. (2010); Kgonothi et al. (2024); Yildiz et al. (2025)
MVD	HWBL, OD, US, US+OD	10‐30	‐ Low ΔE (3.2) and higher β‐carotene retention (80%) than CHAD.	Yan et al. (2013); Lagnika et al. (2021)
MWD+CHAD	–	2	‐ Reduced β‐carotene content (‐79.64%) and TPC (‐30.50%).	Tüfekçi and Özkal (2023)
MWD+IRD	Sodium metabisulphite	5	‐ high β‐carotene retention (80.46%), reduced L*, high ΔE (48.95), increased TPC and high bulk density microstructure.	Kgonothi et al. (2024)
MWSD	SBL, HWBL	10	‐ Reduced drying time (100‐120°C), blanching reduced L*(38.5%), increased ΔE (71.2%), β‐carotene retention (80%).	Yan et al. (2013); Liu et al. (2014)
IRD+FD	US	3.5	‐ Increases β‐carotene content by 4–42%, porous matrix formation.	(Wu et al. 2020; Jiang et al. 2025)
IRD+CHAD	‐	4 – 36	‐ Better color attributes and non‐uniform heating. ‐ Controlled RH and velocity improved drying uniformity coefficient (95.9%), increased TPC (179%).	Onwude et al. (2018); Onwude et al. (2021)
OD+CHAD	–	NR	‐ Improves color, texture, and has better consumer acceptability.	Michalichen et al. (2019)
US+SJR	–	3	‐ at 40°C it reduced drying time (68%), high TPC, high β‐carotene levels, L* and b* value.	Yildiz et al. (2025)

**Abbreviations**: MWSD = microwave assisted spouted drying, NR = not reported, SJR = slot jet reattachment.

### 3.1 Sun and Solar Drying

Open air sun drying (OSD), involves exposing the product directly to the sun without the use of any equipment (Pravitha et al. 2024). In SP drying, research studies have highlighted the limitations of OSD, such as extended drying periods, which can be up to 7 days, due to the low drying rates (Watson et al. 2024). Furthermore, significant contamination by bacteria and fungi has been observed (Watson et al. 2024), along with the darkening of SP (Onyenwigwe et al. 2023). This is because the drying process relies on climatic conditions, which are usually at low drying air temperatures (41°C) and high relative humidity (52%) (Onyenwigwe et al. 2023; Pravitha et al. 2024; Watson et al. 2024). To address the challenges of OSD, solar drying (SD) technologies have been explored as an alternative. Recent advancements in SP drying have focused on cabinet‐type dryers, including the development of an indirect cabinet dryer with enhanced natural ventilation via a solar venturi (Gasa et al. 2022a), a mixed mode dryer with natural convection (Onyenwigwe et al. 2023), a forced convection dryer (Sakouvogui et al. 2023) and an indirect dryer with forced convection and surplus heat storage, using phase change (PCM) material (Abdelkader et al. 2024). Onyenwigwe et al. (2023) found that the SD can save up to 12 h of drying time. A forced convention solar dryer reduced the drying time to 2 days (Sakouvogui et al. 2023), while using a solar venturi to enhance the air flow, a naturally ventilated dryer resulted in a drying time of 13 h (Gasa et al. 2022a). Contrarily, an advanced forced convection dryer with a PCM, namely, paraffin, which could extend the drying process by 5.5 h beyond the sunshine hours shortened the drying time to 52 h (Abdelkader et al. 2024). The dryer was reported to have a high thermal efficiency of 97.2%. The long drying time may have been attributed to the relatively lower drying air temperature (47.3°C). According to Beigi (2016), the thermal efficiency and drying temperature have an inverse relationship. Therefore, although the dryer had high efficiency, it could not achieve a high enough temperature to improve the drying rate. Typically, the SD dryers increase the ambient air temperature by 7 to 8.2°C (Gasa et al. 2022a; Sakouvogui et al. 2023). As a result, the temperature is a significant contributing factor to SD performance. However, research studies on SP drying often neglect the RH and velocity as contributing factors. While SD presents numerous advantages over OSD, challenges such as initial investment and operational costs, may impede its widespread adoption (Venkateswarlu and Reddy 2024). Furthermore, there is no evidence of superior quality retention. For example, Gasa et al. (2022a) found that SD led to dark colored SP. Similarly, Onyenwigwe et al. (2023) noted darker colored samples, especially in blanched SP. Nonetheless, there are long‐term benefits in terms of CO_2_ mitigation potential (12.42 tons per year) (Onyenwigwe et al. 2023) and 20.25 tons, over an anticipated 35‐year lifespan (Abdelkader et al. 2024). Therefore, optimizing SD technologies is essential to achieve a synergistic on the drying time, drying conditions, and quality parameters.

### 3.2 Convective Hot‐Air Drying

**FIGURE 4:**
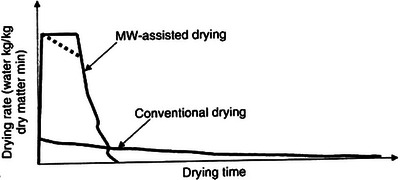
Drying rate curves of microwave (MW)‐assisted drying versus the conventional drying techniques (Wang et al. 2014).

In producing SP flour, convective hot‐air drying (CHAD) is the commonly used technique for the effect of drying air temperature and velocity. Temperatures between 40 and 80°C and velocities between 1 and 4.5 m.s^−1^ have been used, and it was observed that the drying often occurs in the falling rate period (Falade and Solademi 2010; Tayyab Rashid et al. 2022). However, CHAD is associated with relatively long drying times, compared to IR and MW, because it can take between 4 and 28 h to reach a safe storage moisture content (Liu et al. 2017). Additionally, high specific energy consumption (SEC) of 337.79 kWh.kg^−1^ has been reported (Onwude et al. 2018), as well as high values of total color change (ΔE = 29) and reductions in TPC (total phenolic contents) and β‐carotene content have been reported (Moloto et al. 2021; Tayyab Rashid et al. 2022). Hence, recent studies have focused on identifying the optimal drying conditions for energy efficiency and quality retention. These techniques can dry SP at a rate that is twice that of CHAD (Onwude et al. 2019) and reduce the drying time up to six times (Yan et al. 2013), depending on the method used (Jiang et al. 2025). In their 2018 study, Onwude et al. (2018) found that combined IR and CHAD reduced the drying time by 86% compared to using CHAD alone. The combination of IRD and CHAD facilitates heat production within the product, which is simultaneously transferred to the external environment along with the hot‐air transfer from the product surface, resulting in a uniform drying process (Chia et al. 2022). MWD intensifies the heat and mass transfer phenomena at the surface of the product, resulting in an increased drying rate of the CHAD method (Figure 4). While these emerging techniques show promise, arguments persist regarding combined drying techniques, citing limited penetration depth, non‐uniform heating, and textural damage to product, amongst others (Bhattacharjee et al. 2024; Jiang et al. 2025). Hence, further studies are needed to comparatively assess these techniques to gain an in‐depth understanding of their energy consumption, heating characteristics, and quality retention of SP.

### 3.3 Fluidized Bed Drying

Fluidized bed drying, particularly in the form of spouted bed drying (SBD) is used to dry small cubes (0.1–1 cm^2^) of SP to produce flour. The increasing attention in SBD for drying SP is due to the simple equipment structure (Rezende et al. 2024), low investment cost (Rashid et al. 2022a; Huang et al. 2023), low working pressure, and excellent heat and mass transfer characteristics (Du et al. 2023). Costa et al. (2015) observed that it is suitable for thermosensitive materials, such as SP, because the average temperature of the particles is lower than that of the drying air. In drying sweet potatoes, pure SBD, SBD with inert particles, and the use of SBD with other drying methods have been explored. Among these, the impact of drying temperature on drying characteristics and quality has been extensively studied. Literature revealed that drying air temperatures ranging from 45°C to 120°C have been examined. Rezende et al. (2024), found that using SBD to dry SP at higher drying temperatures results in greater heat transfer, which increases the rate of water migration from the product to the surface. It also increases the specific heat and diffusivity (Huang et al. 2023), allowing the drying time to be reduced by up to three times (Thao and Noomhorm 2011). In addition, microwave enhanced spouted bed drying (MSBD) has a drying time that is seven times shorter than CHAD (Yan et al. 2013), whilst pure SBD can reduce the drying time of IRD by 97% (Thao and Noomhorm 2011). High effective moisture diffusivity, reaching up to 7.26 × 10^−7^ m.s^−2^, has been recorded. In SP drying, microwave is emerging as one of the promising methods to combine with SBD. Yan et al. (2013) and Huang et al. (2023), mentioned that the pneumatic agitation that occurs during spouted bed drying creates uniform drying, thereby addressing the unevenness often associated with MWD. Furthermore, MWSBD improves the rehydration ability of sweet potatoes, by increasing the porosity in their microstructure (Yan et al. 2013; Rezende et al. 2024). However, when combined with high microwave power (4 W.g^−1^), there is evidence of charring. Thus, there is no definitive evidence that SBD satisfactorily preserves quality. Although some studies report better color and β‐carotene retention than CHD and FD and low ΔE (2.6‐3.7) (Yan et al. 2013). There is still a need for future studies to investigate the quality parameters of different SBD methods in drying SP, including the use of inert particles, which are reported to significantly improve the drying rate. Additionally, key parameters influencing this drying process, such as the spouting velocity, maximum spouted bed, and maximum spouted bed pressure drop (Huang et al. 2023), still need to be explored to determine the optimum parameters.

### 3.4 Freeze Drying

Freeze drying (FD) is a widely used drying technique for sweet potatoes, primarily because it preserves the quality of fresh SP. This is attributed to its ability to preserve color and maintain high rehydration capacity (Yao et al. 2023), while preventing the loss of sensitive nutrients, such as carotenoids and total phenolic contents (TPC) (Bhatkar et al. 2021). However, due to its high operating cost and energy consumption that can reach 527.8 MJ.kg^−1^ (Antal 2023), mainly resulting from its prolonged drying time (Lin et al. 2005; Wu et al. 2020). Research has explored combined drying to improve the SP drying kinetics of FD. These include the application of simultaneous FD with microwave/infrared/vacuum whereby drying is carried out in multistages  where FD is followed by microwave/infrared or vacuum finish, or microwave/infrared/vacuum followed by an FD finish (Nwankwo et al. 2023). These strategies are employed to supply the heat required for sublimation during FD. For example, in their study Lin et al. (2005) proposed a single stage far‐infrared freeze dryer (FIRFD) that reduced the drying time up to 11 hours. A recent study found that a multistage mid‐infrared freeze dryer (MIRFD) at an IR intensity to provide a temperature of 60°C for 5 minutes at the beginning of the drying process reduced the drying time by 51.98% and the SEC by 51.98% compared to the single stage FD technique, which completed drying in 21 h (Antal 2023). Furthermore, combined vacuum and freeze drying reduced the drying time by 24%, compared to FD. Microwave assisted freeze drying (MWFD) at a microwave power of 4 g W.g^−1^ was able to dry SP cubes in 4.5 h. Furthermore, it increased the drying time between 135.42% and 233.77%, compared to microwave vacuum drying (MVD) and MSBD, respectively (Liu et al. 2014). FD drying methods have different effects on the quality of SP. For example, notable total color changes have been observed (ΔE > 2) (Lagnika et al. 2021). Hence, factors such as pre‐drying treatments and slice thickness have been identified to influence the effectiveness of FD techniques (Lin et al. 2005; Kręcisz et al. 2021; Lagnika et al. 2021). However, the management of the process conditions during drying is important. Hence, techniques like microwave assisted pulse fluidized bed drying have been proposed, including the use of pre‐drying treatments. Therefore, the advancements in freeze‐drying technology continue to make it a viable option for high‐quality sweet potato products.

### 3.5 Infrared Drying

The primary benefit of infrared drying (IRD) lies in its nature as a volumetric heating method, where heat penetrates directly into the food, allowing it to be absorbed throughout the product. Additionally, the electromagnetic radiation induces vibrations in water molecules, generating internal heat that facilitates rapid moisture transfer to the surface (Kgonothi et al. 2024). The key parameters that influence the performance of IRD are the infrared power, velocity of air, relative humidity, and slice thickness. Doymaz (2012) found that the drying rate increased 2.57 times when the IR power was increased from 104 W to 167 W, while increasing the slice thickness to 8 mm increased the drying time by 108%. A stepwise reduction of RH and increasing the velocity from 1 m.s^−1^ at the beginning of drying up to 4 m.s^−1^ at the end of drying has been found to improve the drying time by 34.48%, compared to constant speed IRD, which haslower energy consumption (4.8 kWh versus 7.9 kWh) (Jiang et al. 2025). Sequential two‐stage CHAD and IRD reduces the drying time up to 78% (Antal 2023). Polat et al. (2022) found that intermittent IRD drying at high power (500 W) yields lower color changes (ΔE = 15) and improves the rehydration capacity of SP. Similarly, intermittent IRD+HAD found relativley low ΔE values (17.15‐26.48) (Onwude et al. 2019). Furthermore, the combination of IRD and MWD showed significant drying time reduction, compared to CHAD (72%), IRD (44%) and MWD (16%). Pre‐drying treatments have been found to enhance IRD, through inactivation of polyphenol oxidase, which causes browning (Song et al. 2021). Ultrasound pre‐drying treatment has been used to trigger rapid heat and mass transfer of moisture for reduction of the drying time (Tayyab Rashid et al. 2022). For example, the use of ultrasound at 40 kHz reduced the drying time by up to 60%, however did not yield better color (ΔE = 29.78). Generally, the synergistic effect of IRD in reducing the drying time, reducing energy, and quality is dependent on several factors, including optimal combination of drying techniques and pre‐drying treatments. Optimizing these factors can lead to significant improvements in drying efficiency and product quality.

### 3.6 Microwave Drying

A notable trend in microwave drying (MWD) of SP is in combined drying and optimizing the microwave drying parameters. This optimization involves determining the ideal temperature and microwave power to minimize the drying time, save energy, and preserve quality. Several studies have found that increasing the microwave power reduces the drying time of SP slices. For instance, a drying time of 25 min was recorded at high microwave power of 900 W (Lee et al. 2019), while 200 min was achieved at 697 W (Abano 2020) and a shorter drying time of 6 min was observed for MWD at 850 W (Marzuki et al. 2021). The electromagnetic waves from the microwave increases the liquid diffusion rate due to the formation of a porous structure during drying (Abano 2020). Tüfekçi and Özkal (2023) observed up to a 91% decrease in the drying time when a single stage CHAD+MWD were performed at 70°C and 180 W, compared to 50°C and 90 W. This study employed response surface methodology (RSM) to optimize the drying conditions, and concluded that a drying temperature of 54.3°C and microwave power of 101.97 W, which dried SP in 71 min were optimal conditions to reduce the drying time and preserve quality. Sobowale et al. (2022) concluded that a two‐stage CHAD+MWD process at 100 W can achieve a drying efficiency of up to 82%. The highest energy consumption of 9.09 × 10^−3^ J.kg^−1^ was observed at 300 W microwave power. Combined drying techniques increase the drying rate, due to rapid, volumetric heating, resulting in increased heat and mass transfer (Marzuki et al. 2021). According to Sui et al. (2019), MVD is one of the emerging drying technologies in drying sweet potatoes. This technique is beneficial for products with active ingredients and heat sensitive components because it further accelerates the drying rate and enhances the transportation of the heat and moisture inside the drying products, while the vacuum enables moisture to evaporate at a lower temperature. Monteiro et al. (2020) used a MVD with a PID temperature control to adjust the microwave power, maintaining a temperature of 60°C within the dryer. Without temperature control it was found that MVD took 83 min whereas where there was temperature control the drying time was 17 min. This study demonstrated that in the absence of power control, noticeable scorching of SP occurred. Hybrid MW‐based techniques are said to preserve quality better. For example, MWVD and MWSB were able to retain 80% of β‐carotene, and lower ΔE values between 2.6 and 3.7 have been reported (Yan et al. 2013). Contrarily, Tüfekçi and Özkal (2023) found that with combined MWD and CHAD, β‐carotene losses reached 84%, with the highest losses occurring at elevated temperatures and microwave power. Combining physical pre‐treatments of ultrasound and osmotic dehydration is a promising technique (Lagnika et al. 2021). While these advancements in MWD present significant benefits, challenges remain in balancing efficiency with quality preservation.

### 3.7 Other Drying Methods

Explosive puffing is a drying method where food is exposed to high temperature (100°C) and pressure (0.4 Mpa) to evaporate the moisture (Xie et al. 2024). In SP drying, it produces puffed snacks and powder. Although beneficial for nutritional quality retention (Kaur et al. 2023), it is often combined with other methods, mainly at intermediate stages (Chen et al. 2017), because it cannot be carried out directly for high moisture content products (Kaur et al. 2023). Using radio frequency explosive puffing at 1500 W on pre‐gelatinized PFSP starch with 60% moisture content, the chips produced had the lowest ΔE (8.67), hardness (752 gf) and highest rehydration ratio (3.42), compared to CHAD, MWD, and VFD (Xie et al. 2024). Osmotic dehydration is a drying method that plays a critical role in stabilizing carotenoids in sweet potato (Oladejo et al. 2017) and facilitates the breakdown of cells, which improves the bio accessibility of carotenoids (Oladejo and Ma 2016). However, to acquire maximum moisture loss during drying, it is often facilitated by the use of pre‐drying methods, including ultrasound (Oladejo and Ma 2016), and in some instances it is used as a pre‐drying treatment (Yan et al. 2013). In conclusion, while combined drying yields promising results, future research should concentrate on optimizing these dryers by considering the key factors that influence the drying process, including the drying air temperature, velocity, slice thickness, and IRD/MW power.

## 4 Factors Influencing Sweet Potato Drying

### 4.1 Drying Conditions

The drying process of SP is significantly affected by conditions, including the drying air temperature, air velocity, and relative humidity (RH). Most experimental studies were conducted in air temperatures between 50 and 80°C and concluded that a high drying air temperature saves time. For example, Doymaz (2011) observed a 28% decrease in drying time when temperature was increased from 50 to 90°C in CHAD. Gonçalves et al. (2023) also observed a faster decrease in moisture loss as the temperature increased from 60 to 80°C. Similarly, with IRD increasing the temperature to 80°C shortened the drying time significantly (Rashid et al. 2019). Contrarily, using the CHAD technique, Oke et al. (2020) found that a temperature of 40°C resulted in longer drying periods. High drying air temperature improves the mass transfer coefficient, increasing the rate of drying (Jiang et al. 2025). Therefore, the drying air temperature and drying time are inversely related, due to the increased moisture gradient. A lower hot‐air temperature (CHAD) of 40°C at a higher air velocity of 4.5 m.s^−1^, reduced the drying time by 20 min (Kgonothi et al. 2024). However, Delfiya et al. (2022) mentioned that faster air movement (velocity > 1.5m.s^−1^) results in a reduced drying rate and increased drying time because it creates a cooling effect on the product and reduces the product temperature. Therefore, the drying air velocity in the range of 1‐1.5 m.s^−1^ is recommended (Pravitha et al. 2024). New developments in SP drying include RH and air velocity control. Studies on combined drying indicate that maintaining an air temperature of 60°C and step‐up the velocity (1 m.s^−1^ to 4 m.s^−1^) and step‐down RH (50% to 5%) can significantly (*p* < 0.05) reduce the drying time of CHAD + IRD by 34.48% (Jiang et al. 2025). Where MWD and IRD were used, the power settings, exposure times, and distance of the sample from the infrared bulb are factors to consider because the drying rate has a positive correlation with power increase (Lee et al. 2019; An et al. 2024). Generally, combined drying methods that include MWD or IRD, improves the drying efficiency and yield a better‐quality product. These are dielectric‐based drying techniques that use electromagnetic waves of frequency between 1.3 × 10^6^ and 2.5×10 Hz to interact with the internal layers of the product (Jin et al. 2018; Fathi et al. 2022). When the frequency interacts with the bipolar water molecules within the product, it repeatedly rotates, thus creating heat throughout the product due to friction between the water molecules. This intensifies the moisture evaporation and transfer to the outer surface of the product (Fathi et al. 2022). Sweet potato drying research focuses more on advanced drying methods, with limited studies on the changes that occur in different drying conditions.

### 4.2 Slice Thickness

The slice thickness is a critical factor that affects the overall drying process. Previous studies conducted drying experiments with a slice thickness between 3 and 9 mm (Tayyab Rashid et al. 2022; Abdelkader et al. 2024; Ezeoha et al. 2024; Kgonothi et al. 2024; Šovljanski et al. 2024), with an exception of 10–30 mm studied by Ezeoha et al. (2024). Thicker slices increase drying time (Savas 2022), energy costs and affect quality parameters (Pravitha et al. 2024). Hence, a slice thickness > 9 mm is not recommended in SP drying because it creates a difficulty in the removal of moisture, due to increased moisture travel distance before evaporating and reduced surface area to volume ratio (Ezeoha et al. 2024). Therefore, it takes longer to reach a safe storage moisture content. Also, IRD has an effective penetration thickness of 0.5–4 mm, thus cannot be used for thicker slices (Jiang et al. 2025). Korese and Achaglinkame (2024) observed a 50% increase in drying time when the slice thickness was increased from 3 mm to 5 mm. This is caused by the increased moisture diffusivity, D_eff_ (internal moisture mass transfer characteristics consisting of all mass transport mechanisms), because of the concentration of moisture in the core of the thicker slices (Abano 2020; Delfiya et al. 2022; Korese and Achaglinkame 2024). Contrarily, Gasa et al. (2022a) observed a higher effective D_eff_ for the 3 mm SP slice thickness (2.19 × 10^−8^–6.3 × 10^−9^ m^2^.s^−1^), compared to 7 mm slices (1.02 × 10^−8^–3.32 × 10^−9^ m^2^.s^−1^). Research suggests that the D_eff_ variation with slice thickness is due to case hardening. It generally occurs quicker in thinner slices, restricting moisture transfer during drying, hence reducing the moisture diffusivity (Delfiya et al. 2022; Pravitha et al. 2024). Case hardening is negligible in thicker slices, hence increasing the moisture transfer and D_eff_. High drying air temperature is a contributing factor and could explain contradictory findings by (Gasa et al. 2022a). Therefore, future studies should examine the theoretical background of case hardening and its effects on the D_eff_ of SP at different slice thicknesses and temperatures, velocity, and RH. Lastly, several studies have recommended to dry SP with a slice thickness of 3 mm (Abano 2020; Korese and Achaglinkame 2024) to prevent nutrient losses.

### 4.3 The Use of Pre‐Drying Treatments Prior to Drying

**FIGURE 5:**
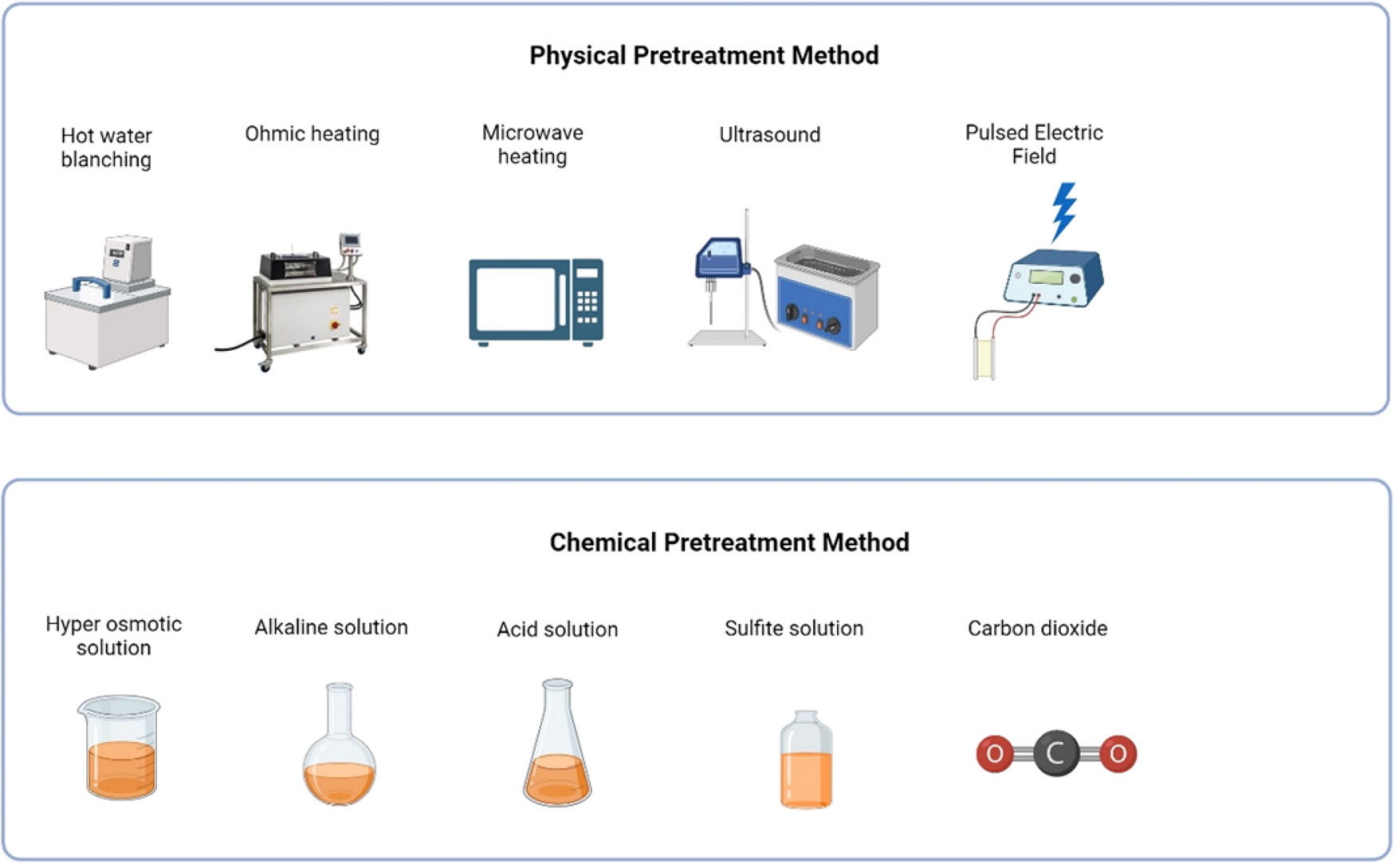
The physical and chemical pre‐drying methods used prior to SP drying (created with Biorender.com).

Chemical, physical, and thermal pre‐drying treatments, as shown in Figure 5, are used to reduce the drying process time and enhance the quality of the dried product. These techniques aim to eliminate intercellular air from SP tissues and soften the texture to facilitate drying. Pre‐drying treatments are assumed to induce higher water activation energies, to start the drying process of SP (Dinrifo 2012). Hot water blanching (HWB) and steam blanching (SB) are widely used thermal pre‐drying techniques as documented in previous studies (Marzuki et al. 2021; Ayonga et al. 2023; Korese and Achaglinkame 2024; Wang et al. 2024). Contrary to general knowledge, blanching can decrease drying rates, leading to prolonged drying periods (Abano 2020; Chinenye et al. 2022). This depends on the slice thickness, blanching period, and temperature. For example, extending the blanching duration from one to three minutes increased the drying time by 2.3% (Chinenye et al. 2022) and 33% (Xiao et al. 2009). For slices thicker than 6 mm, the D_eff_ increased with an increase in the blanching period (Abano 2020). Furthermore, blanched SP samples were reported to require a high amount of energy to initiate the drying process (E_a_), compared to untreated samples (Korese and Achaglinkame 2024). This may be caused by starch gelatinization, making the product less permeable to moisture transfer (Abano 2020). As a result, thicker SP slices subjected to longer blanching periods required a longer drying time. Wang et al. (2024) further explained that HWB of 5 mm slices for two minutes results in high permeability, andthere is evidence of case hardening when the blanching period is extended to four minutes. Lagnika et al. (2021), suggest blanching is useful in long‐term storage because the water gain that occurs during blanching provides a constant moisture content and water activity during storage by providing stability to SP. Hence, future studies need to validate the cell permeability and hardness and overall effect of blanching on SP during drying.

In recent years, drying has been enhanced by energy saving physical pre‐drying techniques, such as ultrasound and microwave. They form micro‐channels in the product, which enhances the water mobility (mass transfer) outside of the product, during drying (Vithu et al. 2020). The use of ultrasound at a higher frequency of 60 kHz did not significantly reduce the drying time compared to the lower frequencies of 20 to 40 kHz (Rashid et al. 2019). A low frequency reduces a need for high drying air temperature. For example, at 80°C and 60 kHz frequency, the drying time increased by 20%, whereas at 20 kHz and 40 kHz it decreased by 20% and 40%, respectively. Contrarily, Infante et al. (2021) found that an ultrasound pre‐drying treatment of 25 kHz and drying air temperature of 50°C resulted in a longer drying period, compared to 70°C. This study raised the pre‐treatment conditions, as a possible influencing factor to the drying time. Similarly, microwave pre‐drying treatment has displayed a higher rate of moisture transfer through SP, compared to blanching (Abano 2020). Furthermore, increasing the microwave power from 385 W to 697 W and extending the exposure period from two to four minutes increased the drying rate and reduced the drying time. The D_eff_ for microwave assisted hot air drying ranged from 1.5 × 10^−9^ to 4.4 × 10^−9^ m^2^.s^−1^ while blanching had values between 1.1 × 10^−10^ and 7.9 × 10^−10^ m^2^.s^−1^. The higher D_eff_ in microwave pre‐treated samples can be attributed to the increased thermal energy and activity of water molecules in the SP samples. Radio‐frequency, freeze‐thawing, and osmotic dehydration are pre‐drying treatments that also facilitate the moisture migration to the surface of SP samples (Osae et al. 2024). The pre‐drying treatment conditions, such as temperature, frequency, power, and the treatment period are the main factors that influence the results. While these factors are essential for optimizing the drying processes, it is also important to consider the trade‐offs between drying efficiency and the retention of nutritional quality. Advanced pretreatment methods offer promising solutions, but their implementation must be tailored to specific product requirements.

## 5 Changes to Physicochemical Properties That Occur During Drying

This section will discuss the surface color, β‐carotene content, TPC, and microstructure as some of the physicochemical properties that are affected by the drying process. The effect of drying on these parameters is summarized, as shown in Figure 7 and Table 2.

**FIGURE 6:**
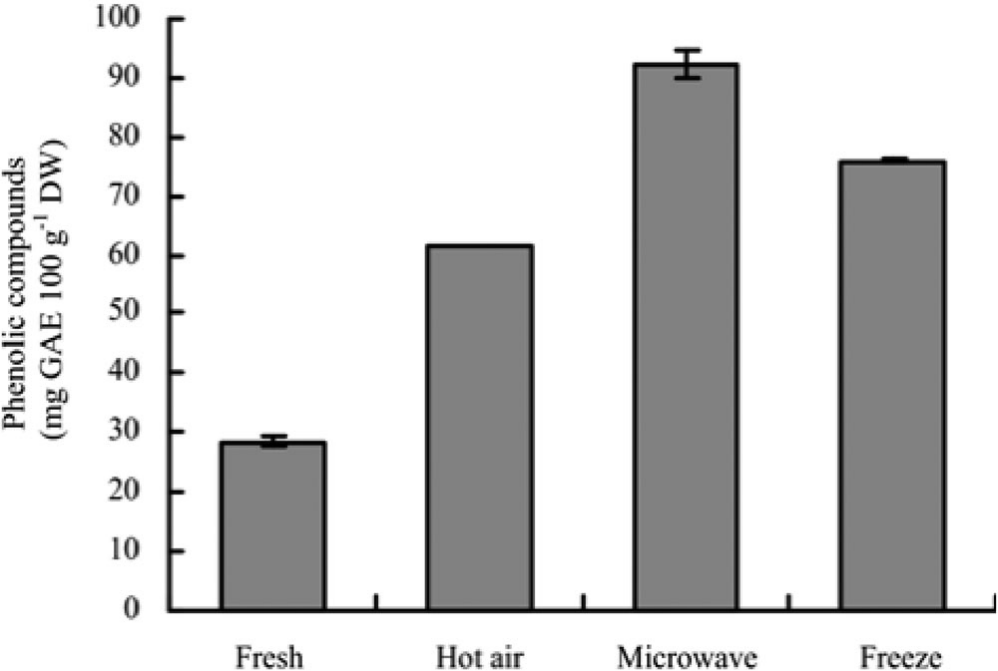
A comparison of the TPC of fresh SP and various drying methods (Jing et al. 2010).

**FIGURE 7:**
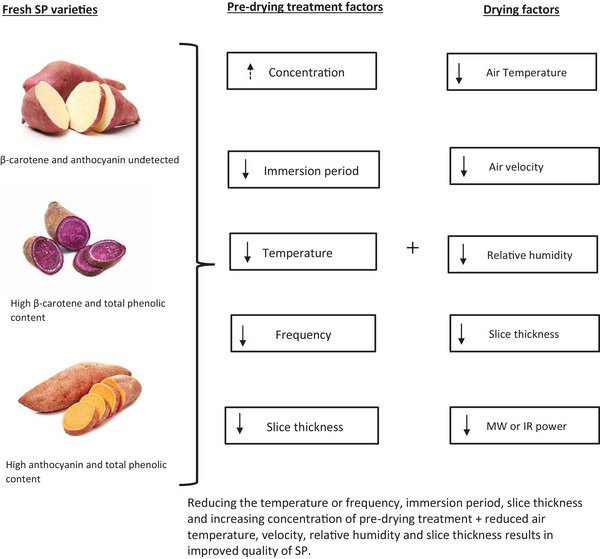
An illustration of the effects of drying on SP (↑ = increase, ↓ = decrease).

### 5.1 Surface Color

Color is the main physical parameter that draws consumers to food products and is an indicator of nutrient availability (Korese and Achaglinkame 2024). SP color is attributed to the quantities of pigments, including carotenoids (yellow, orange, and red color) and anthocyanins (red and blue) (Bhattacharjee et al. 2024). Color changes are caused by oxidation that occurs during drying and are described by the CIELAB color space coordinates L* (lightness), a*(redness), b* (yellowness), and ΔE (total color change). Drying significantly (*p* < 0.005) affects these parameters (Kręcisz et al. 2021; Kgonothi et al. 2024) and generally increases the L*, a*, and b* color parameters of SP (Savas 2022; Jusoh et al. 2025). Furthermore, the ΔE of dried SP has been reported to be visible (ΔE >2) and significantly higher than that of fresh samples (Kręcisz et al. 2021; Lagnika et al. 2021; Kgonothi et al. 2024; Korese and Achaglinkame 2024). CHAD reduces the L*, and increases a*, b* and ΔE values (Onwude et al. 2018; Marzuki et al. 2021). Confirming the statement that dried samples are generally less bright, more reddish, and more yellowish than fresh samples, due to the degradation of color pigments. For example, Onwude et al. (2018) observed a significant decrease (*p ≤* 0.05) in L* value up to 41%. Significantly higher ΔE values (34.24) are observed for drying techniques that have longer drying periods, such as CHAD (Kgonothi et al. 2024). Contrarily, this study also found that these techniques can reduce the a* and b* parameters of OFSP, with a decrease of 46.86% and 60% compared with fresh samples, respectively. This is caused by a slow drying period of that causes Maillard reactions before the safe storage moisture content is reached (Bhattacharjee et al. 2024). Combined drying techniques, including MWD + IRD and CHAD + IRD have achieved lower ΔE values between 28.10 and 37, due to reduced drying times (Kgonothi et al. 2024; Jiang et al. 2025).

As discussed above, the drying method influences the color changes. Other factors like drying conditions (air temperature, velocity, and power), slice thickness, and the pre‐drying treatment are the main causes of color parameter fluctuations (Onwude et al. 2019; Lagnika et al. 2021; Ezeoha et al. 2024; Jusoh et al. 2025). Sturm et al. (2023) and Infante et al. (2021) confirmed that drying at temperatures of 40°C and 70°C had higher ΔE than drying at temperatures of 60°C. Drying air temperature above 70°C and oxygen presence in CHAD activates polyphenol oxidase (PPO) and browning due to thermal degradation of the natural pigments (Onwude et al. 2018; Marzuki et al. 2021). Liu et al. (2014), highlighted that PPO's are active at room temperature and only degrade at high temperatures. Ezeoha et al. (2024) found that increasing the temperature to 85°C significantly reduced the b* value by 4.79% for 10 mm SP slices and by 35.59% for 30 mm slices. This confirms that both the drying air temperature and slice thickness have a synergistic effect on SP during drying. Other drying conditions, such as the infrared power, influence the color parameters, for example, Wu et al. (2020) found that when using infrared power of 600 W, the color changes were not significant at ΔE = 4. An extension of the distance of 12 to 18 cm exhibited a substantial rise of ΔE up to 100% (Song et al. 2021).

The use of pre‐drying treatment prior to drying has been found to assist drying techniques in retaining the color parameters (Marzuki et al. 2021; Ayonga et al. 2023; Osae et al. 2024). Physical pre‐drying treatments like ultrasound and osmotic dehydration significantly lower ΔE (12.55), compared to the conventional blanching technique (ΔE = 38.87) (Wu et al. 2020; Lagnika et al. 2021; Curayag and Dizon 2023). Ultrasound disrupts cellular membranes without causing substantial modification to the chemical processes, increasing SP lightness (Kręcisz et al. 2021; Pravitha et al. 2024). Sugar‐based pre‐drying treatments, such as glucose solution and citric acid are more effective in inhibiting enzymatic browning reactions than ultrasound (Tayyab Rashid et al. 2020). Contrarily, Curayag and Dizon (2023) found significant browning when using 1% CA for PFSP drying. Indicating that the concentration of the pre‐drying treatment could be a factor that influences the color preservation properties of sugar‐based drying treatment. Furthermore, Zhao et al. (2016) revealed that carbon maceration is effective in reducing enzymatic browning by decreasing the duration of the drying process. With physical and chemical pre‐drying treatment, the immersion period plays a critical role in the effectiveness of the methods. For example, Karacabey et al. (2023) found that extending the blanching period from 2 to 4 min resulted in an increase in the L* value. The drying method and pre‐drying treatment combination also influence the color changes, for example, carbon maceration pre‐drying treatment in conjunction with intermittent MWD leads to lower ΔE (14.8). The combination of BL with radio frequency heating has also been found to significantly reduce ΔE by up to 40% (Jiang et al. 2020). In conclusion, there is a syngenetic effect of the drying method and its settings (temperature, power, etc.), slice thickness and pre‐drying treatment conditions (e.g., immersion time, frequency, and temperature) on the color parameters. Future studies should integrate all factors, including SP variety, to draw conclusions on this parameter's influence. In hindsight, because the PFSP and OFSP varieties contain high levels of anthocyanin and phenolic compounds, enzymatic browning easily occurs (Liu et al. 2014).

### 5.2 β‐Carotene

The β‐carotene content positively correlates with L*, a*, and b* color parameters (Kgonothi et al. 2024). Whereas Wu et al. (2020) claimed that only the b* value relates to the β‐carotene content of SP. Therefore, the factors mentioned in 5.1 influences the β‐carotene content. Ruttarattanamongkol et al. (2016), substantiate this in their study by concluding that conventional drying techniques like CHAD can significantly degrade β‐carotene in OFSP. β‐carotene degradation is lower at 55°C than 65°C (Kuyu et al. 2018). At 80°C, the β‐carotene content of OFSP decreased 22.7‐fold compared to 8.3‐fold at 70°C. Hence, Sturm et al. (2023) and Zhang et al. (2018) recommended a drying air temperature of 60°C to preserve β‐carotene. Using combined drying techniques (MWD+IRD) has been found to improve the β‐carotene retention (85%) better than CHAD (23.61%), FD (43.13%), IR (66.04%), and MW (80.46%) (Kgonothi et al. 2024).

Pre‐drying treatments generally increase the β‐carotene content. Chemical pre‐drying treatments have been found to retain or increase β‐carotene levels, attributed to reduced activity of enzymes, such as peroxides and lipoxygenases (Kuyu et al. 2018). For example, Ahmed et al. (2010) showed that the utilization of sodium hydrogen sulfite did not significantly affect the β‐carotene content when SP was in CHAD. Carbon maceration results in a β‐carotene retention of approximately 62.7% when applied prior to intermittent MWD and 41.8% before continuous MWD (Zhao et al. 2016). The inclusion of carbon (CO_2_) during carbon maceration hinders oxidation reactions, protecting the β‐carotene from deterioration. Ultrasound pre‐drying treatment enhanced the β‐carotene content up to 500% during CHAD (Rashid et al. 2022b). Wu et al. (2020) found that the β‐carotene content increased from 4% to 42% when US was applied prior to IRD. Osmotic dehydration showed no statistically significant difference in β‐carotene content. When steam and microwave BL were employed, the retention of β‐carotene decreased considerably to 5.93 mg.100g^−1^ and 4.19 mg.100 g^−1^. The β‐carotene retention of novel BL techniques, such as conveyor belt catalytic infrared, was considered to be comparatively greater, reaching up to 133%, in contrast to HWBL (109%) (Song et al. 2021). The β‐carotene content is positively correlated with an increase in the slice thickness (Clifford et al. 2014; Ezeoha et al. 2024). Clifford et al. (2014) found that increasing the slice thickness from 2 mm to 4 mm results in β‐carotene content losses up to 86%. Therefore, the use of pre‐drying treatments and combined drying methods is necessary to preserve β‐carotene during SP drying.

### 5.3 Total Phenolic Content

The total phenolic content (TPC) of SP is greatly impacted by the variety, drying method, conditions, pre‐drying treatments, and extraction method. Fresh and dried PSP varieties have a significantly (*p* < 0.05) higher TPC than OFSP and WFSP varieties (Ruttarattanamongkol et al. 2016; Johnson et al. 2022). Genetic factors also play a significant role in the formation of secondary metabolites, including phenolics (Kuyu et al. 2018). However, the effect of drying on these varieties depends on the drying method used. Ruttarattanamongkol et al. (2016) mentioned that thermal processes enhance the TPC of SP. Contrarily, studies on CHAD found it negatively affects phenolic stability, due to chemical and enzymatic degradation. CHAD has resulted in significant (*p* < 0.05) TPC reduction of 76%, 45.5%–33% and 28.4%–67.72%, compared to CHAD+MVD (Lagnika et al. 2021), MWD (Ahmed et al. 2010; Jing et al. 2010), and FD (Ahmed et al. 2010; Lagnika et al. 2021), respectively (Figure 5). MWD can increase the TPC by threefold compared to CHAD, with up to a 225% increase reported for SP (Jing et al. 2010), as illustrated in Figure 6. TPC reduction in thermal drying techniques depends on the air temperature; between 65°C and 70°C the TPC reduces up to 53% (Ruttarattanamongkol et al. 2016; Kuyu et al. 2018; Savas 2022). Contrarily, at 55°C, TPC can increase up to 115%, further enhanced by the use of pre‐drying treatments prior to drying.

Blanching pre‐treatment reduces the TPC levels during drying (24%), due to the heat or leaching of SP tissues into the water (Lagnika et al. 2021). Citric acid at 1% w/v can lead to a 53.5% decline in TPC values, compared to 3% w/v (Kuyu et al. 2018). However, with osmotic dehydration (OD), a high concentration (20% w/v) of the osmotic solution increased TPC up to 91.98% (Tayyab Rashid et al. 2020). High concentrations of chemical solutions likely reduce the oxidation rate of polyphenol compounds. Where physical pre‐drying treatments, such as ultrasound, were used at a moderate frequency (40 kHz), TPC increased up to 60%, compared to 20 kHz (Rashid et al. 2019). However, a high concentration of glucose (20% w/v) resulted in TPC values that were higher that that of ultrasound alone and ultrasound + glucose treatments (Tayyab Rashid et al. 2020). A combination blanching + ultrasound assisted OD, prior to CHAD + MWD, retained the TPC by 66.9 % (Lagnika et al. 2021). However, a higher TPC increase of up to 247% was observed when carbonic maceration was used prior to MWD. To retain TPC, consider optimal temperature (60°C), US frequency (40 kHz), and high chemical concentrations. Drying and pre‐drying in the absence of oxygen are promising to significantly increase TPC.

### 5.4 Microstructure Changes

The most important phenomenon that occurs during drying is the cell contraction that prompts a significant change in the product structure and allows the release of water (Tayyab Rashid et al. 2020). Fresh SP microstructure revealed a well‐organized cellular structure with neat, uniform cellular organization and distinct visibility of the cell wall and starch granules. The drying method, conditions, slice thickness, and pre‐drying treatments significantly affect the SP's cellular structure during drying. Dried SP typically has a porous structure with ruptured cells and destructed intercellular spaces (Tayyab Rashid et al. 2020). Thermal drying techniques, such as CHAD and IRD, create high bulk density, creating a less porous structure than FD, which creates a sponge structure (Savas 2022; Kgonothi et al. 2024). Increased porosity facilitates moisture diffusion, reduces the drying time, and improves product quality. High drying air temperatures (80°C) result in a rough structure with voids. Using combined drying techniques (CHAD+IRD and MWD+IRD) resulted in a more uniform cellular structure and greater pores, compared to individual drying techniques (Onwude et al. 2018; Kgonothi et al. 2024), indicating intercellular stress that reduces the drying rate (Rashid et al. 2019; Savas 2022). Contrarily, the use of combined drying methods with pre‐drying treatments creates smaller micropores and less intercellular space breakage, increasing diffusion and drying rate (Rashid et al. 2019). For example, the use of medium frequency US (40 kHz) produces a smooth structure with smaller micropores than 60 kHz. However, Liu et al. (2017) indicate that a high ultrasound frequency causes larger pore formation, which induces cyclic stretching and contraction of the intercellular space, known as the “sponge effect”, which enhances moisture transfer. The formation of the pores is through acoustic cavitation, and Wu et al. (2020) indicated that the pore size parameter is also influenced by the ultrasound power intensity. Glucose pre‐drying treatment creates less damage to the intercellular spaces, which increases diffusion and reduce the drying time, compared to ultrasound. Additionally, chemical pre‐drying treatments, such as sodium hydrogen sulfite have been reported to destroy starch granules of SP because the alkali conditions weaken the structure of starch by breaking the inter‐and intra‐molecular hydrogen bonds within the starch chain (Wootton and Manatsathit 1984). Combined pre‐drying treatments, such as ultrasound + OD are reported to smaller pore sizes than the traditional HWBL technique, enabling better moisture transfer (Lagnika et al. 2021). BL creates a gel‐like structure in dried SP with low porosity (Lagnika et al. 2021), leading to the disappearance of a granular structure due to starch gelatinization (Lagnika et al. 2018). The extent of the starch gelatinization is influenced by the immersion period (Trancoso‐Reyes et al. 2016). When compared to HWBL, SBL and VSPD increased the number of large cell walls, which enlarge the cells for better moisture transfer during drying. Therefore, combined pre‐drying treatment provides a porous structure, by enlarging and increasing the rate of moisture transfer during drying.

## 6 Conclusions and Future Prospects

In recent years, research efforts have focused on breeding SP with high nutrition (e.g., β‐carotene and anthocyanin) and drought tolerance. This aims to sustain SP production as a food nutrition and security crop, due to increased consumption that is supported by functional food development. These efforts have driven research in SP drying technologies. This review found that conventional drying techniques, such as CHAD, are popular due to simplicity. However, they damage the cellular structure of SP, causing non‐uniform drying, longer drying periods and reduced physicochemical properties. The drying process effectiveness is influenced by the method, conditions, slice thickness, SP variety, and pre‐drying treatments. Suggested parameters include a slice thickness > 3 mm, temperature > 60°C, RH∼5–50%, velocity > 1.5 m.s^−1^. Additionally, a combination of physical and chemical pre‐drying treatments effectively reduces the drying time and preserves quality. However, with most drying techniques, there is notable (e.g., ΔE > 2) color changes that occur. Recent studies have used combined drying methods/hybrid drying and combined pre‐drying techniques (e.g., US + OD) to optimize the drying process and reduce the color changes. Although these advanced techniques yield better product quality, future research should prioritize:

- Collaborative strategies across regions to improve SP yield for sustainable production, focusing on widely adaptable genotypes and value‐added products that are preferred by consumers.
- Optimization of drying techniques considering all process parameters, using machine learning techniques like computer vision for quality analysis can simplify comparative analysis of process conditions and their effect on quality.
- Development of energy‐efficient, sustainable drying methods that preserve quality.

Lastly, ensuring sustainability and adoption of optimized drying techniques that preserve quality could increase the demand for SP products (e.g., medicinal), driving production.

## Author Contributions

**Khuthadzo Mugodo**: conceptualization, investigation, funding acquisition, writing–review and editing, writing–original draft, data curation, formal analysis, visualization. **Tilahun Seyoum Workneh**: project administration, supervision, writing–review and editing. **Sunette Laurie**: validation, supervision, writing–review and editing. **Naushad Emmambux**: writing–review and editing, validation, supervision.

## Conflicts of Interest

The authors declare no conflicts of interest.

## Supporting information




**jfds70458‐sup‐0001‐tableS1.docx**: Table S1
