# Supplementary material for: Different Drying Techniques and Their Impact on Physicochemical Properties of Sweet Potato: A Review
Source: J Food Sci. 2025 Aug 11;90(8):e70458. doi: 10.1111/1750-3841.70458 (PMC12336766; doi:10.1111/1750-3841.70458)
Supplement: Supplementary file 1 — jfds70458‐sup‐0001‐tableS1.docx: Table S1 [file JFDS-90-0-s001.docx]

Table S1. A summary of the physicochemical properties of fresh sweet potato varieties

| **Composition** | **Orange-fleshed** | **White fleshed** | **Cream fleshed** | **Yellow-fleshed** | **Purple-fleshed** | **References** |
| --- | --- | --- | --- | --- | --- | --- |
| **Dry matter content (%)** | 14-32 | NR | NR | NR | NR | Gurmu et al. (2024)^g^ |
|  | 18.9-27.1 | NR | 17.4-25. | NR | NR | Laurie et al. (2015) ^a, b^ |
|  | 17.5-33.1 | 18.1-35 | NR | 25-35 | 25.1 | Wu et al. (2008)^c^ |
|  | 31.4-36.5 | 39.9-41.6 | 35.9-39.3 | 32.3 | NR | Namutebi et al. (2004)^d^ |
| **Moisture content (%, db)** | 70.95 | NR | NR | NR | NR | Alam (2021)^f^ |
|  | 72.47-82.40 | NR | 71.87-74.77 | 74.30 | NR | Laurie et al. (2022)^a^ |
|  | 62.78-64.03 | 70.51-72.45 | NR | 68.58 -72.73 | NR | Dako et al. (2016)^g^ |
|  | 69.42 | NR | NR | NR | 73 | Rodrigues et al. (2016)^e^ |
|  | 65.1-70.4 | NR | NR | NR | NR | Nicanuru et al. (2015)^h^ |
|  | NR | 62.58 | NR | 62.78-64.03 | NR | Rose and Vasanthakaalam (2011)^k^ |
| **Starch (g.g^-1^)** | 65.41 | NR | NR | NR | 103.70 | Rodrigues et al. (2016)^e^ |
|  | 95.7-167.7 | NR | 164.3 | 156.7-163.3 | NR | Laurie et al. (2022)^a^ |
|  | NR | 67.5-61.5% | NR | 45.8-53.1 | 53-55.9 | Guo et al. (2019)^c^ |
|  | 98.5% | 99% | NR | 97% | 99% | Soison et al. (2015)^f^ |
| **Protein (g.100g^-1^/%, db)** | NR | NR | NR | NR | 19.12-28.81 | Ngcobo et al. (2024)^a, b^ |
|  | 1.91-5.83 | NR | NR | NR | NR | Alam (2021)^g^ |
|  | 0.8-1.20% | NR | 1.07-1.53% | 1.10% | NR | Laurie et al. (2022)^a^ |
|  | 2.48-2.84 | 3.46-4.60 | NR | 4.41-6.50 | NR | Dako et al. (2016)^g^ |
|  | 0.1 | 0.1 | NR | 0.4 | 0.1 | Soison et al. (2015)^f^ |
|  | 3.69 | NR | NR | NR | 5.70 | Rodrigues et al. (2016)^e^ |
|  | 4.21-4.4% | 2.72-4.88% | NR | NR | 5.52-5.90% | (Kim et al., 2011)^g^ |
|  | 1.44-2.50 | 2.45 | 1.47 | NR | NR | Lyimo et al. (2010)^h^ |
| **Fat (g.100g^-1^)** | NR | NR | NR | NR | 0.47-0.83 | Ngcobo et al. (2024)^a, b^ |
|  | 0.17-0.30 | NR | NR | NR | NR | Alam (2021)^f^ |
|  | 0.10 | NR | ND | 0.17 | NR | Laurie et al. (2022)^a^ |
|  | 1.00-1.12 | 0.53-0.72 | NR | 0.49-0.66 | NR | Dako et al. (2016)^g^ |
|  | 0.42 | NR | NR | NR | 0.42 | Rodrigues et al. (2016)^e^ |
|  | 0.03-0.95 | 0.14 | 0.16 | NR | NR | Lyimo et al. (2010)^h^ |
|  | NR | NR | NR | NR | 2.51-2.86 | Ngcobo et al. (2024)^a, b^ |
| **Dietary Fiber (g.100g^-1^)** | 3.83-4.52 | 2.94-5.24 | NR | 3.59-6.65 | NR | Dako et al. (2016)^g^ |
|  | 2.37-4.07 | NR | 2.87-3.37 | 3.57 | NR | Laurie et al. (2022)^a^ |
|  | 3.68 | NR | NR | NR | 2.57 | Rodrigues et al. (2016)^e^ |
| **Beta-carotene (mg.100g^-1^)** | 4.3.06-21.40 | NR | NR | NR | NR | Gurmu et al. (2024)^g^ |
|  | 38.7-82.93 | NR | 0 | 25.61 | NR | Laurie et al. (2022)^a^ |
|  | 121.6 | NR | NR | 15.7 | 12 | Kuan et al. (2016)^i, j^ |
|  | 300-530 | 0-125 | NR | 14.7-28.5 | 2.8-3 | Kim et al. (2015)^h^ |
|  | 24.2-73.9 | NR | NR | NR | NR | Nicanuru et al. (2015)^h^ |
|  | 5091-16456 μg.100g^-1^, fw | NR | 14-134  μg.100g^-1^, fw | NR | NR | Laurie et al. (2012)^a, b^ |
|  | 4.66-4.99 | ND | NR | NR | NR | Rose and Vasanthakaalam (2011)^k^ |
|  | 34.6-83.3 | NR | NR | 4.3-9.7 | NR | Shih et al. (2009)^l^ |
|  | 24.1-231.1 | 5.2-7.8 | NR | 123-128.9 | 0.60 | Wu et al. (2008)^c^ |
| **Anthocyanin content (μg. g^-1^)** | NR | ND | NR | ND | 57.9 | Kuan et al. (2016)^i^ |
|  | 2.98 | NR | NR | NR | 7.14 | Kim et al. (2015)^h^ |
|  | 4-8.5 | NR | NR | 2-3.6 | NR | Shih et al. (2009)^l^ |

Abbreviations: NR = not reported**,** ND = not detected**.** dm = dry weight basis**,** fw = fresh weight basis, superscript represents the country of origin of SP varieties whose data are presented in the table, a = South Africa, b = United Sates of America, c =China, d = Uganda, e = Brazil, f = Bangladesh, g = Ethiopia, h = Tanzania, i = Japan, j = Indonesia, k = Rwanda, l = Taiwan

**Supplementary Table References**

Alam, M. K. (2021). A comprehensive review of sweet potato (Ipomoea batatas [L.] Lam): Revisiting the associated health benefits. *Trends in Food Science & Technology*, *115*(2021), 512-529. <https://doi.org/https://doi.org/10.1016/j.tifs.2021.07.001>

Dako, E., Retta, N., & Desse, G. (2016). Comparison of three sweet potato (Ipomoea batatas (L.) Lam) varieties on nutritional and anti-nutritional factors. *Global Journal of Science Frontier Research: D Agriculture and Veterinary*, *16*(4), 1-11.

Guo, K., Liu, T., Xu, A., Zhang, L., Bian, X., & Wei, C. (2019). Structural and functional properties of starches from root tubers of white, yellow, and purple sweet potatoes. *Food Hydrocolloids*, *89*, 829-836.

Gurmu, F., Mekonnen, B., & Habete, B. (2024). Evaluation of orange-fleshed sweetpotato genotypes for root yield and yield-related traits in South and Northern parts of Ethiopia. *Cogent Food & Agriculture*, *10*(1), 2376204. <https://doi.org/10.1080/23311932.2024.2376204>

Kim, H. J., Park, W. S., Bae, J.-Y., Kang, S. Y., Yang, M. H., Lee, S., Lee, H.-S., Kwak, S.-S., & Ahn, M.-J. (2015). Variations in the carotenoid and anthocyanin contents of Korean cultural varieties and home-processed sweet potatoes. *Journal of Food Composition and Analysis*, *41*(2015), 188-193. <https://doi.org/https://doi.org/10.1016/j.jfca.2015.01.012>

Kim, J.-M., Park, S.-J., Lee, C.-S., Ren, C., Kim, S.-S., & Shin, M. (2011). Functional properties of different Korean sweet potato varieties. *Food Science and Biotechnology*, *20*(2011), 1501-1507.

Kuan, L. Y., Thoo, Y. Y., & Siow, L. F. (2016). Bioactive components, ABTS radical scavenging capacity and physical stability of orange, yellow and purple sweet potato (I pomoea batatas) powder processed by convection‐or vacuum‐drying methods. *International journal of food science & technology*, *51*(3), 700-709.

Laurie, S., Faber, M., Adebola, P., & Belete, A. (2015). Biofortification of sweet potato for food and nutrition security in South Africa. *Food Research International*, *76*(October 2015), 962-970.

Laurie, S., Van Jaarsveld, P., Faber, M., Philpott, M., & Labuschagne, M. (2012). Trans-β-carotene, selected mineral content and potential nutritional contribution of 12 sweetpotato varieties. *Journal of Food Composition and Analysis*, *27*(2), 151-159.

Laurie, S. M., Bairu, M. W., & Laurie, R. N. (2022). Analysis of the Nutritional Composition and Drought Tolerance Traits of Sweet Potato: Selection Criteria for Breeding Lines. *Plants*, *11*(14), 1804.

Lyimo, M., Gimbi, D., & Kihinga, T. (2010). Effect of processing methods on nutrient contents of six sweet potato varieties grown in lake zone of Tanzania. *Tanzania Journal of Agricultural Sciences*, *10*(1), 55-61.

Namutebi, A., Natabirwa, H., Lemaga, B., Kapinga, R., Matovu, M., Tumwegamire, S., Nsumba, J., & Ocom, J. (2004). Long-term storage of sweetpotato by small-scale farmers through improved post harvest technologies. *Uganda Journal of Agricultural Sciences*, *9*(1), 914-922.

Ngcobo, A., Mianda, S. M., Seke, F., Sunette, L. M., & Sivakumar, D. (2024). Phytonutritional composition and antioxidant properties of southern African, purple-fleshed sweet potato (Ipomoea batatas (L.) Lam.) Storage Roots. *Antioxidants*, *13*(3), 338-359.

Nicanuru, C., Laswai, H., & Sila, D. (2015). Effect of sun-drying on nutrient content of orange fleshed sweet potato tubers in Tanzania. *Sky Journal of Food Science*, *4*(7), 91-101.

Rodrigues, N. d. R., Barbosa Junior, J., & Barbosa, M. (2016). Determination of physico-chemical composition, nutritional facts and technological quality of organic orange and purple-fleshed sweet potatoes and its flours. *International food research journal*, *23*(5).

Rose, I. M., & Vasanthakaalam, H. (2011). Comparison of the nutrient composition of four sweet potato varieties cultivated in Rwanda. *American journal of food and nutrition*, *1*(1), 34-38.

Shih, M.-C., Kuo, C.-C., & Chiang, W. (2009). Effects of drying and extrusion on colour, chemical composition, antioxidant activities and mitogenic response of spleen lymphocytes of sweet potatoes. *Food chemistry*, *117*(1), 114-121.

Soison, B., Jangchud, K., Jangchud, A., Harnsilawat, T., & Piyachomkwan, K. (2015). Characterization of starch in relation to flesh colors of sweet potato varieties. *International Food Research Journal*, *22*(6), 2302.

Wu, X., Sun, C., Yang, L., Zeng, G., Liu, Z., & Li, Y. (2008). β-carotene content in sweet potato varieties from China and the effect of preparation on β-carotene retention in the Yanshu No. 5. *Innovative Food Science & Emerging Technologies*, *9*(4), 581-586.
